# Multi‐Omics Analysis Reveals That Aβ42‐ Me3 Co‐Aggregation Induces Mitophagy Blockade in Early Stages of Alzheimer's Disease

**DOI:** 10.1002/mco2.70984

**Published:** 2026-09-08

**Authors:** Wenjie Zhu, Yuanyuan Zhang, Pengjie Li, Yingying Zhong, Yawei Lin, Xin Liu

**Affiliations:** ^1^ The Key Laboratory for Biomedical Photonics of MOE At Wuhan National Laboratory for Optoelectronics‐Hubei Bioinformatics and Molecular Imaging Key Laboratory Systems Biology Theme Department of Biomedical Engineering College of Life Science and Technology Huazhong University of Science and Technology Wuhan China; ^2^ School of Chemistry Chemical Engineering and Life Sciences Wuhan University of Technology Wuhan China

**Keywords:** β‐amyloid, Alzheimer's disease, malic enzyme 3, mitophagy, proteomics

## Abstract

Mitochondrial dysfunction is one of the earliest pathological features of Alzheimer's disease (AD), preceding overt neurodegeneration and cognitive decline. Amyloid‐β (Aβ) accumulation has long been considered a central pathogenic event in AD, yet how Aβ toxicity is mechanistically linked to mitochondrial impairment during early disease stages remains incompletely understood. To address this, we combined multi‐omics with in vivo and in vitro genetic interventions. Here, we show that malic enzyme 3 (Me3) links Aβ aggregation to mitochondrial dysfunction in APP/PS1 mice and neuronal cells. At ultra‐early and early AD stages (3 and 6 months), Me3 was upregulated and accumulated within mitochondria, where it colocalized with Aβ42 and physically interacted with it, an association linked to oxidative stress and impaired mitophagy. Knockdown of Me3 reduced mitochondrial reactive oxygen species, improved mitochondrial morphology, and alleviated mitophagy defects in both cellular and mouse models, with statistical significance across these functional measurements (*p* < 0.05). These results suggest that Me3 functions not only as a metabolic responder to Aβ‐associated stress but also as a contributor to early mitochondrial pathology. By identifying the Aβ‐Me3 axis, this study provides mechanistic insight into early mitochondrial dysfunction in AD, while further validation in human AD samples remains necessary.

## Graphical abstract

Using multi‐omics techniques, including proteomics, targeted metabolomics, and structural proteomics, we observed that the early stage of Alzheimer's disease (AD) progression was accompanied by mitochondrial dysfunction aggravation, the entry of excessive Aβ into the mitochondria, and the co‐aggregation of Aβ and Me3 protein, thus inducing tricarboxylic acid (TCA) cycle disorder and reactive oxygen species increase, in turn causing mitochondrial dysfunction and mitophagy disruption.

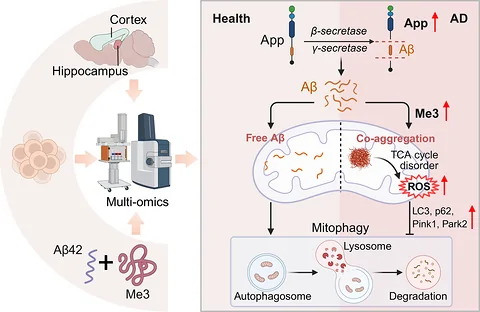

## 1 Introduction

Alzheimer's disease (AD) is a neurodegenerative disorder characterized by progressive cognitive impairment, posing a huge socioeconomic burden [1, 2]. The pathogenic mechanisms of AD are complex, and effective therapies remain lacking [2, 3]. Numerous studies have demonstrated that pathological changes in AD begin 5–15 years before the onset of clinical manifestations [1, 4, 5, 6, 7]. Accurate early screening and diagnosis enable intervention at the initial stage of AD, potentially contributing to AD prevention and AD progression slowing down.

One of the primary pathological hallmarks of AD is the deposition of amyloid‐β (Aβ) in the brain [8]. Aβ, the main component of amyloid plaques, is generated from amyloid precursor protein (APP) cleaved by specific enzymes (such as β and γ secretases) [8, 9]. Excessive Aβ accumulation in particular brain regions, particularly between neurons, will result in plaque formation [9]. It is generally acknowledged that the entorhinal cortex (EC), a component of the brain's limbic system adjacent to the hippocampus, serves as the initial region where Aβ plaques deposit [10, 11, 12]. Aβ deposition not only impairs interneuronal communication but also triggers neuronal apoptosis, thereby severely compromising cognitive functions such as learning and memory [13, 14, 15].

Additionally, mitochondrial dysfunction is another prominent early pathological characteristic of AD, with the accumulation of damaged mitochondria in early stages [16, 17]. As the “powerhouses” of cells, mitochondria are responsible for producing most of the cellular energy in the form of adenosine triphosphate (ATP). Neurons in the brains of AD patients exhibit significant mitochondrial dysfunction and structural abnormalities at early stages, including a reduction in cristae count, elevated reactive oxygen species (ROS) levels, and decreased activity of respiratory chain complexes [18]. Excessive ROS can promote Aβ production, reduce ATP generation, and disrupt mitochondrial membrane permeability [18, 19]. In AD patients, this ROS accumulation further damages neurons, leading to cortical and hippocampal atrophy, mitochondrial functional impairment, and ultimately causing neuronal damage [20].

As a mechanism for eliminating damaged mitochondria, mitophagy is obviously impaired in the brains of AD patients, resulting in the accumulation of damaged mitochondria, further exacerbating neuronal oxidative stress and metabolic disorder [21]. Mitophagic dysfunction has been reported to be closely associated with the aggregation of Aβ42 [22]. Aβ42 can interfere with the PINK1/Parkin signaling pathway, and it can also inhibit the fusion of autophagosomes with lysosomes, thereby aggravating mitophagic dysfunction [23, 24]. Me3, a malic enzyme located in the mitochondrial matrix, modulates the tricarboxylic acid (TCA) cycle and is involved in mitochondrial quality control [25]. Under physiological conditions, Me3 expression is typically lower than that of other malic enzyme isozymes (such as Me1 and Me2), playing a regulatory role in specialized metabolic pathways despite no significant contribution to basal metabolism [26]. However, under certain stresses, Me3 is often significantly upregulated to meet aberrant cellular metabolic demands [27]. Although the roles of Me3 in cancer have been investigated [27, 28, 29], few studies have been conducted to explore its functions in AD.

The APP/PS1 mouse model co‐expressing mutant human APP and presenilin‐1 protein exhibits accelerated Aβ plaque deposition, reflecting key early amyloid pathologenesis of AD. These mice tend to develop Aβ plaques at age of approximately 6 months, providing a well‐characterized temporal framework for investigating AD progression and therapeutic interventions [30, 31].

In this study, we conducted proteomic analyses of the hippocampus and cortex of 3‐ and 6‐month‐old APP/PS1 mice. Comparative analyses revealed that the early progression of AD was closely linked to mitochondrial dysfunction. Notably, the Me3 expression was upregulated in all AD groups, consistent with the abnormal TCA detected by target metabolomics. Abnormalities in the morphology and function of mitochondria were confirmed. Co‐aggregation of Aβ and Me3 led to increased ROS levels and impaired mitophagy. Finally, Me3 knockdown lowered ROS and p62 (a protein responsible for autophagy) levels, thus reducing mitochondrial impairment and restoring mitophagy, eventually mitigating AD.

## 2 Results

### 2.1 Proteomics Reveals Mitochondrial Dysfunction in the Early Stages of AD

**FIGURE 1:**
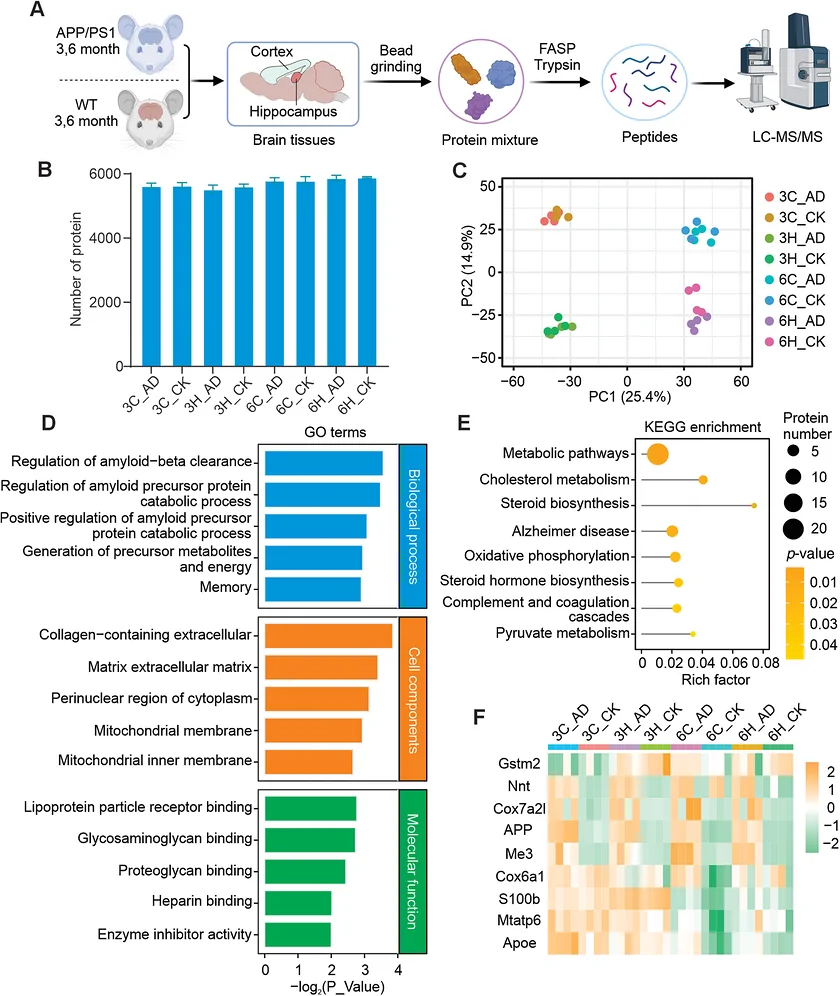
Proteomic profiling of mitochondrial dysfunction in the early stage of AD mice. (A) Schematic workflow of the proteomic experiment. Proteins of cortical and hippocampal tissues from 3‐ and 6‐month‐old APP/PS1 and WT mice were digested using the filter‐aided sample preparation (FASP) protocol and detected by LC‐MS/MS (created with BioRender.com). (B) Number of proteins identified from 4 AD groups (cortex and hippocampus, 3‐ and 6‐month‐old) and 4 CK groups. (C) Principal component analysis (PCA) revealed apparent clustering of genotype (APP/PS1 vs. WT), age, and brain region. (D) Annotated Gene Ontology (GO) terms related to AD and mitophagy in AD mice. (E) Kyoto Encyclopedia of Genes and Genomes (KEGG) pathway enrichment analysis of DEPs in comparison of AD versus healthy mice. DEPs were mainly significantly enriched in cholesterol metabolism, pyruvate metabolism, oxidative phosphorylation, and Alzheimer's disease pathways. Color represents significance (*p* value), and dot size indicates the number of DEPs enriched in pathways. (F) Heatmap of some AD‐related DEPs (mainly including App, Me3, and Cox7a2l) in AD groups versus healthy groups. Orange represents upregulation, and green indicates downregulation.

To systematically characterize the early pathological changes in AD, we collected cortical and hippocampal tissues from APP/PS1 mice (AD groups) and C57BL/6J controls (CK groups) at the ages of 3 and 6 months, yielding eight groups (with four biological replicates per group). Proteomics analysis was conducted on these samples after filter‐aided sample preparation (FASP) (Figure 1A) [32]. A total of 7126 proteins, including 976 mitochondrial proteins, were identified from all the samples (Figure 1B and Figure S1A, Table S1). Correlation analyses demonstrated strong intragroup consistency in protein expression, with an obvious intergroup discrepancy (Figure S1B). The principal component analysis (PCA) of the global proteomic dataset revealed that in early AD, protein expression profiles of APP/PS1 mice and controls were generally close in spite of measurable separation (Figure 1C). In contrast, separation between age groups or brain regions was distinct, suggesting the reliability of our proteomics data.

A total of 161 differentially expressed proteins (DEPs) (fold change [FC] ≥ 1.5, *p* < 0.05) were identified in the comparison of APP/PS1 (AD) versus C57BL/6J (healthy control) mice, of which 30 (18.6%) were mitochondrial proteins (Table S2). Gene Ontology (GO) enrichment analysis revealed three distinct functional categories, including biological process, cellular component, and molecular function. In the biological process category, DEPs were mainly annoted to GO terms such as regulation of amyloid‐beta clearance and generation of precursor metabolites and energy; in the cellular component category, collagen‐containing extracellular matrix and mitochondrial compartments; and in the molecular function category, lipoprotein particle receptor binding and enzyme inhibitor activity (Figure 1D). Additionally, Kyoto Encyclopedia of Genes and Genomes (KEGG) pathway enrichment analysis of DEPs was performed. As shown in Figure 1E, DEPs were mainly enriched in the following AD‐related pathways, such as sterol metabolism, cholesterol metabolism, pyruvate metabolism, AD, and oxidative phosphorylation. Notably, some DEPs exhibited consistently altered expressions; for example, APP, Me3, and Cox7a2l were upregulated relative to four control groups (3‐ and 6‐month‐old mice from cortex and hippocampus tissues) (Figure 1F). Upregulated expression of APP suggested that APP might be a key driver of AD pathogenic processes in APP/PS1 mice. In addition, Me3 and Cox7a2l were both enriched in the mitochondrial pathway, implying that these proteins were likely to be AD‐related.

### 2.2 Mitochondrial Structural Damage Is Exacerbated in 6‐Month‐Old AD Mice

**FIGURE 2:**
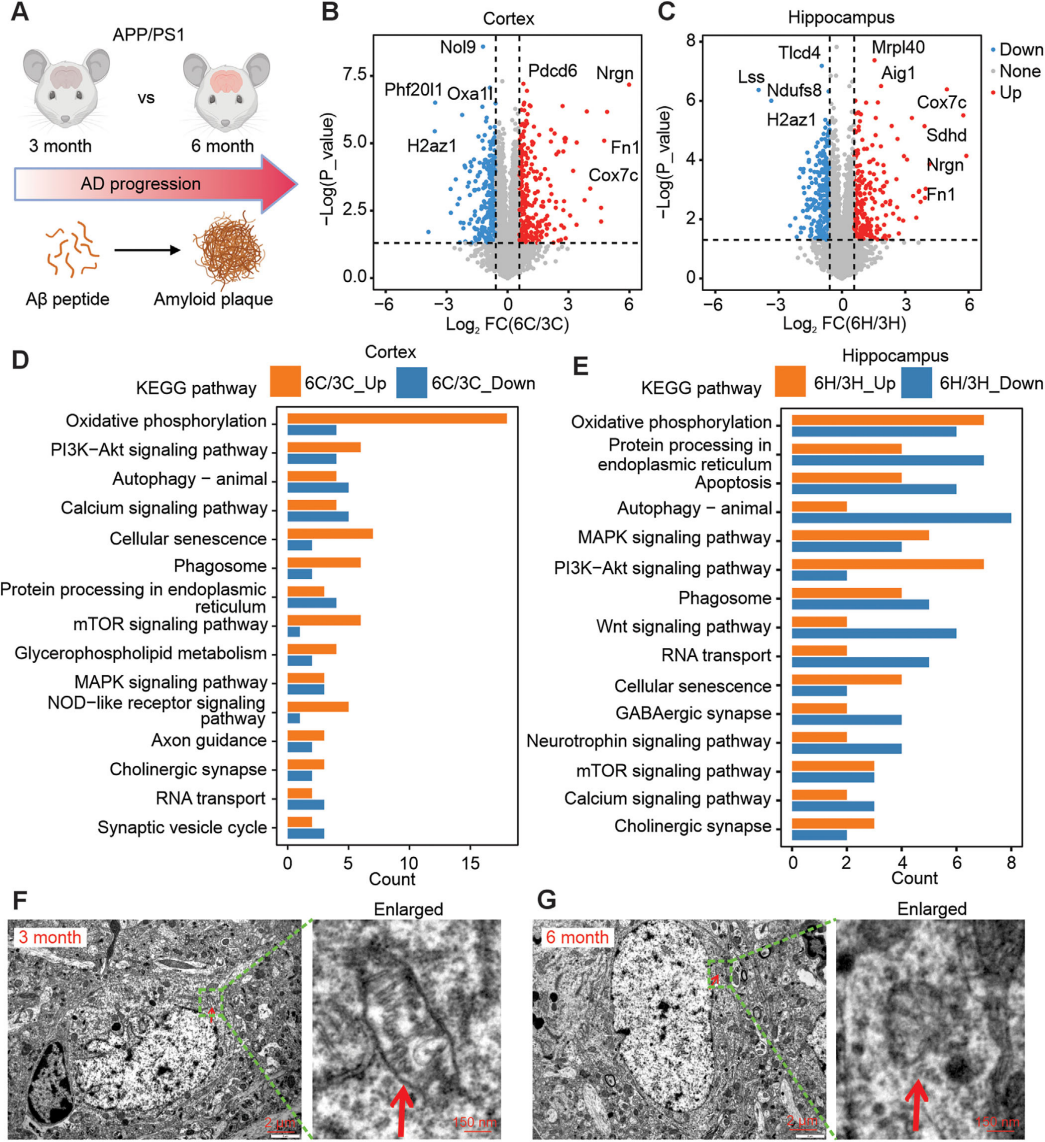
Proteomic analysis reveals progressive mitochondrial dysregulation in AD mice from 3 to 6 months of age. (A) Schematic diagram of the gradual aggregation of Aβ peptides into amyloid plaques in APP/PS1 mice from 3‐month‐old to 6‐month‐old (created with BioRender.com). (B, C) Volcano plot of DEPs in the cortex (B) and hippocampus (C) in comparison of 6‐month‐old versus 3‐month‐old AD mice, respectively. (D, E) AD‐ and mitophagy‐related pathways significantly enriched with DEPs in the cortex (D) and hippocampus (E) by KEGG enrichment analysis. (F, G) TEM images of mitochondrial morphology in the hippocampus of 3‐month‐old (F) and 6‐month‐old (G) AD mice, respectively. The enlarged view revealed mitochondrial dysfunction in AD mice. Scale bars are shown in the TEM images.

Further, we performed a comparison of protein expression levels between 6‐month‐old and 3‐month‐old APP/PS1 mice (Figure 2A). Totally, 936 DEPs were identified in comparison of 6‐month‐old versus 3‐month‐old AD mice, of which 171 DEPs were mitochondrial proteins (Figure 2B,C, Tables S3 and S4). KEGG pathway enrichment analysis revealed that these DEPs were significantly enriched in the AD pathway and mitochondria‐associated pathways. In the cortex (Figure 2D), the top three pathways with the maximum DEPs were the following pathways: oxidative phosphorylation pathway (top 1) was enriched with the largest number of DEPs, of which upregulated DEPs were much more than downregulated ones, whereas PI3K‐Akt signaling pathway (top 2) and autophagy signaling pathway (top 3) were enriched with comparable numbers of upregulated and downregulated DEPs. In the hippocampus (Figure 2E), the oxidative phosphorylation pathway was also top 1 in terms of the number of enriched DEPs, with the number of upregulated DEPs comparable to that of downregulated ones, followed by protein processing in the endoplasmic reticulum (top 2), apoptosis (top 3), and autophagy (top 4) pathways, with more downregulated DEPs than upregulated ones. Notably, the number of downregulated DEPs enriched in the autophagy pathway was far larger than the upregulated ones. Since numerous DEPs were enriched in mitochondria‐related pathways, we further examined mitochondrial morphology in AD mice brain. The transmission electron microscopy (TEM) results showed that some mitochondria exhibited mild swelling, and other mitochondria showed reduced cristae or loss of cristae in the neuronal cytoplasm of hippocampal tissue (Figure 2F,G). Taken together, the above results jointly indicated that neuronal mitochondria were subjected to structural and functional damage.

### 2.3 Targeted Metabolomics Reveals That APP Overexpression Disturbs the TCA Cycle

**FIGURE 3:**
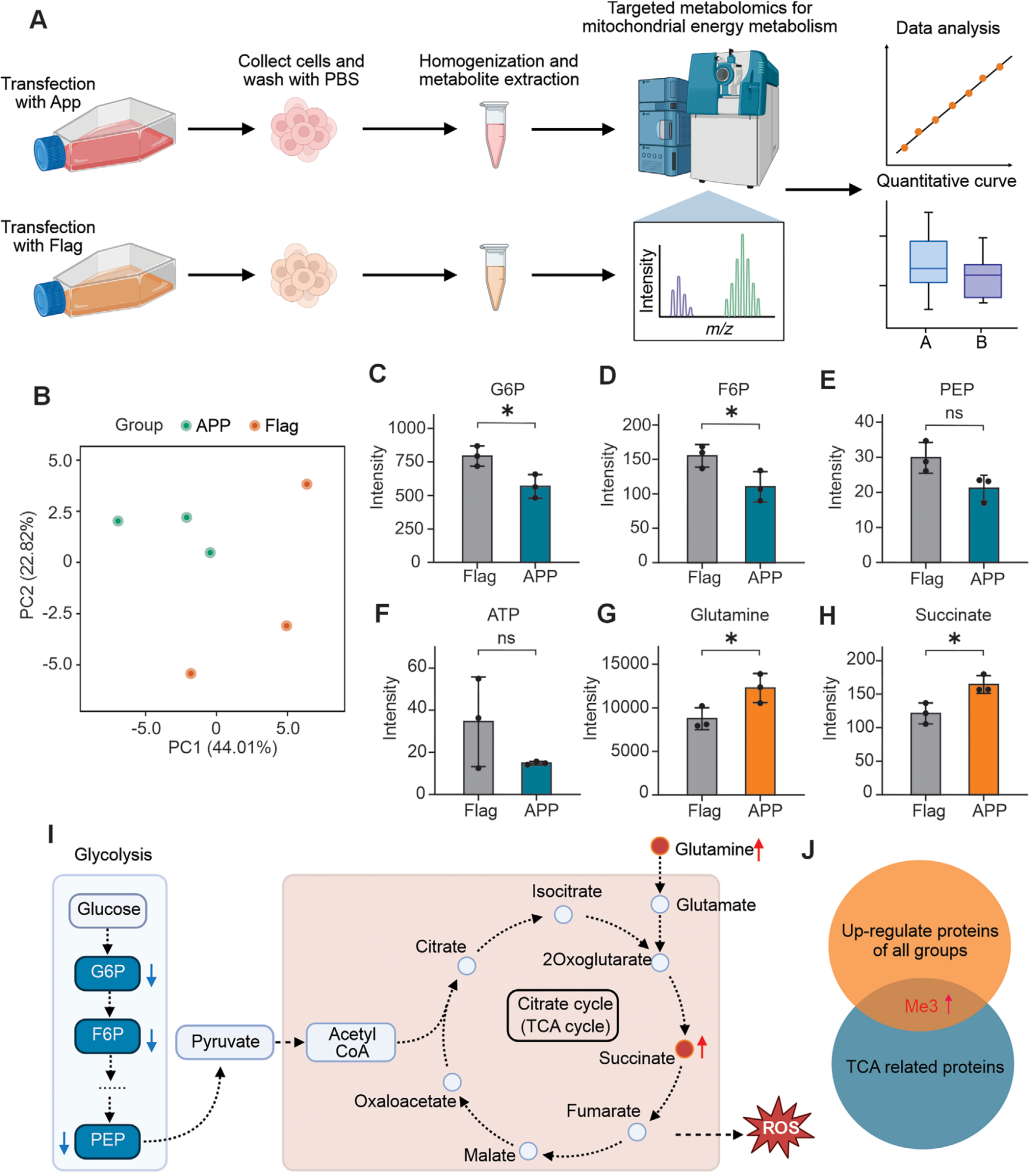
Targeted metabolomic analysis of dysregulation of glycolysis and TCA cycles in APP‐overexpressing cells. (A) Workflow of targeted metabolomics for mitochondrial energy metabolism. HT22 cells were transfected with APP and Flag (control) plasmids, followed by metabolite extraction and LC‐MS/MS analysis (created with BioRender.com). (B) PCA analysis reveals a distinct clustering of APP‐transfected cells and controls. (C–H) Mass intensity of representative metabolites, including D‐glucose‐6‐phosphate (G6P) (C) and D‐fructose‐6‐phosphate (F6P) (D), phosphoenolpyruvate (PEP) (*p* = 0.0587) (E), ATP levels (*p* = 0.1842) (F), glutamine (G), and succinate (H) levels. Data were expressed as mean ± SEM of three biological replicates. Statistical significance was determined by Mann–Whitney test. ns, not significant; *p < 0.05. (I) Schematic diagram of metabolic alterations with glycolysis downregulated (blue arrows) and succinate/glutamine accumulated (red arrows), further driving redox imbalance and ROS production (created with BioRender.com). (J) Venn diagram of DEPs from proteomic analysis and TCA cycle‐related proteins from metabolomic analysis, highlighting Me3 as a consistently upregulated mitochondrial enzyme overlapped in proteomic and metabolomic analyses.

Given that mitochondrial dysfunction and energy metabolism disorder are early AD pathological hallmarks, we investigated metabolites related to mitochondrial energy metabolism through targeted metabolomics analysis. We overexpressed APP in HT22 cells and profiled 55 relevant metabolites via liquid chromatography‐tandem mass spectrometry (LC‐MS/MS) (Figure 3A and Figure S2, Table S5). PCA analysis demonstrated distinct clustering of APP‐overexpressing cells and control cells, indicating a global change in metabolic profiles (Figure 3B). With APP upregulated, the contents of cellular d‐glucose‐6‐phosphate (G6P), d‐fructose‐6‐phosphate (F6P), and phosphoenolpyruvic acid (PEP) were lowered (Figure 3C–E), directly limiting substrate availability for glycolysis. The limited glycolysis, accompanied by a reduction in ATP levels (Figure 3F), further inhibited the generation of pyruvate (a key substrate of the TCA cycle). In contrast, increased cellular glutamine levels (Figure 3G) indicated that APP‐overexpressing cells took up more glutamine and converted it into 2‐oxoglutarate (a TCA cycle intermediate downstream of pyruvate), thereby supplying more intermediates to the TCA cycle and accelerating succinate production. However, succinate accumulated under the limitation of the pyruvate‐related process, indicating the TCA cycle was disrupted (Figure 3H). The accumulated succinate might further promote the production of ROS, ultimately leading to neuronal oxidative damage [33]. Together, these results suggested that TCA cycle dysregulation might act as a key driver of mitochondrial impairment in APP‐overexpressing contexts (Figure 3I).

### 2.4 Aβ Aggregation Colocalized With Me3 and Impaired Mitophagy in Mitochondria

To further explore the linkage between Aβ aggregation and mitochondrial impairment, proteins related to the TCA cycle were screened in our proteomic data. Among TCA‐related protein datasets, only Me3 was consistently upregulated across all four AD groups (Figure 3J). The upregulated expression of Me3 was further observed in Western blot (WB) analysis (Figure S3A,B). In contrast, the other malic enzyme isoforms, Me1 and Me2, showed no significant changes in either the proteomic data from AD mice or the WB analysis of the cellular APP‐overexpression model (Figure S4A–D). This suggested that Me3, rather than Me1 or Me2, may be selectively involved in mitochondrial impairment associated with Aβ aggregation. We next examined Me3 expression under various stress conditions, including H_2_O_2_‐induced oxidative stress, tunicamycin‐induced endoplasmic reticulum (ER) stress, rotenone‐induced mitochondrial impairment, and sodium dichloroacetate (2‐DCA)‐induced TCA cycle stress (Figure S4E). Notably, only 2‐DCA treatment led to a marked increase in Me3 protein levels, indicating that TCA cycle disruption specifically drove Me3 accumulation. We also evaluated Me3 protein stability using cycloheximide (CHX) chase analysis. CHX assay results showed that Me3 protein levels declined more slowly in APP‐overexpressing cells than in negative control (NC) cells, suggesting that APP overexpression delayed Me3 degradation, which may contribute to its accumulation under pathological conditions (Figure S4F).

**FIGURE 4:**
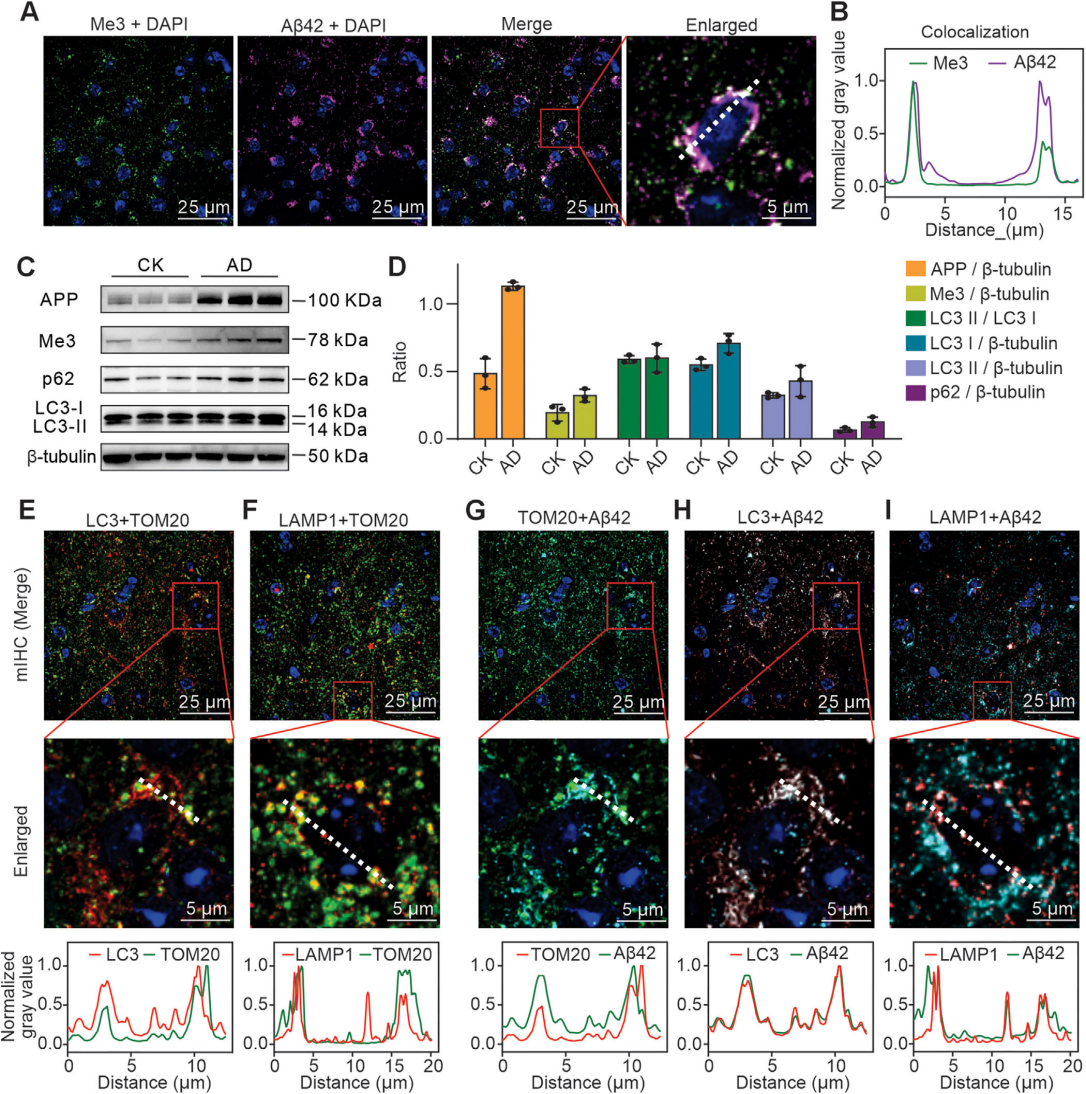
Me3–Aβ42 co‐aggregate and mitophagy impairment in AD mice. (A) mIHC fluorescence signals of Me3 (green) and Aβ (magenta) in AD mice brain tissue. Merged and enlarged views demonstrate prominent colocalization of Me3 and Aβ42. (B) Line‐scan analysis of colocalization of Me3 and Aβ42. (C) Protein level in 3‐month‐old mouse hippocampus tissue by WB analysis. The increased Me3 and p62 levels and conversion of LC3‐I into LC3‐II in AD mice suggested impaired autophagy. (D) Histogram of protein levels. Data were expressed as mean ± SEM of three biological replicates. (E–I) mIHC fluorescence signals and colocalization line‐scan analysis of LC3 (red) and TOM20 (green) (E); LAMP1 (red) and TOM20 (green) (F); TOM20 (green) and Aβ42 (cyan) (G); LC3 (red) and Aβ42 (cyan) (H); LAMP1 (red) and Aβ42 (cyan) (H). Colocalization of LC3 and TOM20 indicated mitophagosome formation, and colocalization of LAMP1 and TOM20 indicated mitophagosome‐lysosome fusion.

To explore the role of Me3 in mitochondrial impairment, we examined the distribution of Aβ and Me3 in the brain tissue of AD mice. The multiplex immunohistochemistry (mIHC) assays revealed that some Me3 exhibited an aggregated distribution and colocalized with Aβ aggregation, indicating a potential co‐aggregation of Aβ42 and Me3 in the early stage of AD (Figure 4A,B). Meanwhile, mitophagy (the key pathway for eliminating dysfunctional mitochondria) was also determined through WB and mIHC assays. The expression of LC3‐I/II (hallmark of autophagy) and p62 in hippocampal tissues was significantly higher in AD mice than in healthy controls, and the ratio of LC3‐II/LC3‐I exhibited no significant changes (Figure 4C,D and Figure S5), suggesting a greater demand for mitophagy and more severe impaired mitophagy in AD. Then, mIHC assays were conducted to observe mitophagy in AD mice directly. The colocalization of LC3 and TOM20 (mitochondrial marker) indicates the initiation of the mitophagic pathway (Figure 4E and Figure S6A), and the colocalization of LAMP1 (lysosomal marker) and TOM20 suggests the fusion of damaged mitochondria with lysosomes for subsequent degradation of mitochondria (Figure 4F and Figure S6B). Notably, we also observed that Aβ42 aggregation colocalized with TOM20, LC3, and LAMP1 (Figure 4G–I and Figure S6A,B), indicating that Aβ42 aggregation intervened in mitophagy and facilitated mitophagy impairment. Collectively, these results suggest that Aβ (generated by cleavage of upregulated APP in AD mice) might co‐aggregate with upregulated Me3 in mitochondria, ultimately impairing mitochondrial function and mitophagy.

### 2.5 Aβ42 Interacts With Me3 to Form Co‐Aggregation in Mitochondria

**FIGURE 5:**
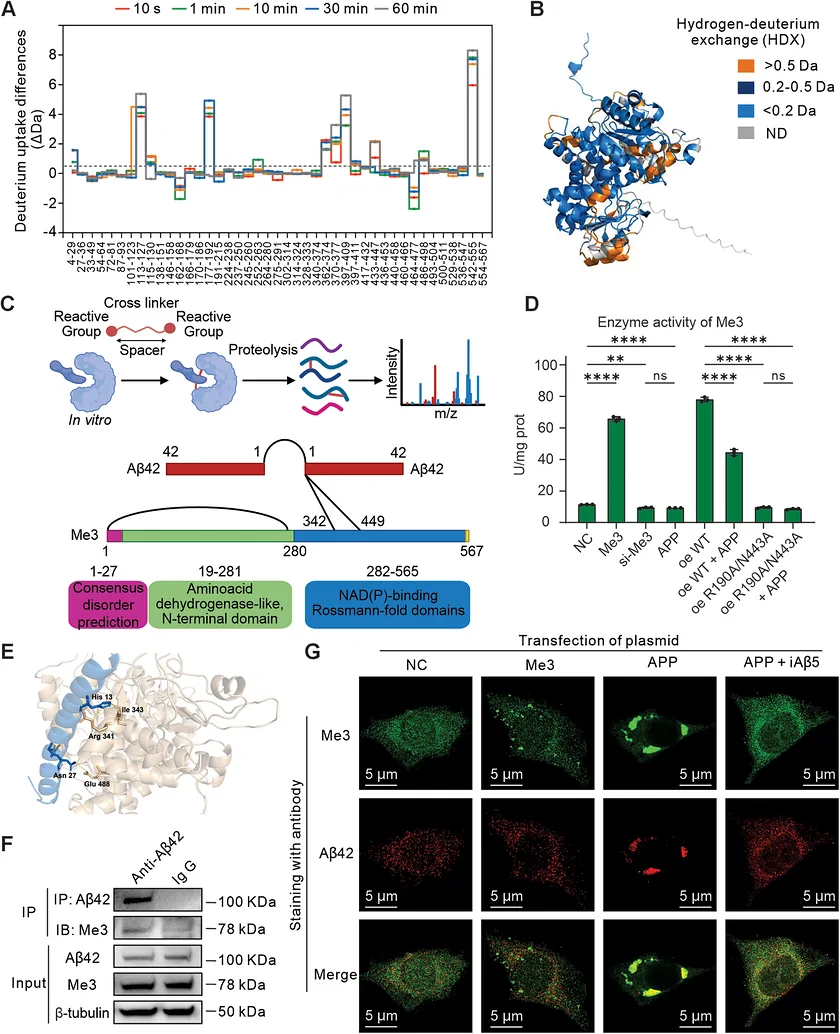
Aβ42 interacts with Me3 to form co‐aggregates in mitochondria. (A) Deuterium uptake differences (ΔDa) between Aβ42‐binding Me3 and unbound Me3. The *X*‐axis lists peptide residue ranges. (B) Structural model of Me3. Different colors represent various hydrogen‐deuterium exchange (HDX) degrees (expressed by Da). (C) Schematic diagram of Me3–Aβ42 interaction by CL‐MS (created with BioRender.com). (D) Quantification of the enzyme activity of Me3. Data were expressed as mean ± SEM of three biological replicates; **p < 0.01; ****p < 0.0001 by Mann–Whitney test. (E) Key residue sites of the interaction between Me3 (Arg341, Ile343, and Glu488) and Aβ42 (His13 and Asn27) simulated by HDOCK. (F) Co‐IP analysis showed the interaction between Aβ42 and Me3. Cell lysates were immunoprecipitated with an anti‐Aβ42 antibody or control IgG, then immunoblotted with anti‐Aβ42 or anti‐Me3 antibody. (G) Immunofluorescence images of HT22 cells in various treatment groups: negative control (NC), Me3‐overexpressing (Me3), APP‐overexpressing (APP), and APP‐overexpressing cells treated with iAβ5 (APP + iAβ5) groups. The row shows antibody staining of Me3 (green) and Aβ42 (red).

To determine whether Aβ42 and Me3 directly interacted, we first performed a hydrogen‐deuterium exchange mass spectrometry (HDX‐MS) assay in vitro with unbound Me3 and Me3 binding with Aβ42 (at a molar ratio of 1:2). This technique measures hydrogen‐to‐deuterium exchange in proteins exposed to D_2_O buffer, with ligand binding shielding the protein surface from solvent and slowing isotopic exchange to reveal interaction sites. After quenching the deuterium exchange process, Me3 was digested with pepsin, and the resulting peptide segments, covering 81.3% of its amino acids, were used to map Aβ42 binding regions to Me3. Upon incubation with Aβ42, deuterium uptake of Me3 exhibited dramatic changes at two regions (HDX1 and HDX2) spanning several peptide segments, with region HDX1 spanning residues 113–127, 177–192, and the other region HDX2 spanning residues 362–374, 370–377, 397–409, 433–447, 486–498, 542–555 (Figure 5A,B and Figure S7). These results indicated that Aβ42 might bind to the HDX1 and HDX2 regions of Me3, thereby reducing their solvent accessibility in the labeling buffer.

To determine specific Aβ42–Me3 binding sites, we cross‐linked the binding regions of Aβ42 and Me3 using bis (sulfosuccinimidyl) suberate (BS3) cross‐linker. After cross‐linking, the cross‐linked peptide fragments were detected by LC‐MS/MS [34]. As a result, we identified two cross‐link residues in Me3, including K342 and K449 (Figure 5C and Figure S8). These two residues are located within the NAD(P)‐binding domain of Me3, suggesting that Aβ42 binding might affect the Me3 catalytic function (Table S6). We also measured Me3 enzymatic activity in Me3‐knockdown cells re‐expressing wild‐type Me3 or the catalytically impaired R190A/N443A mutant. APP overexpression significantly decreased Me3 enzymatic activity compared with NC and attenuated the increase induced by wild‐type Me3 re‐expression, whereas the R190A/N443A mutant displayed markedly reduced activity and showed no further decrease upon APP co‐expression (Figure 5D and Figure S9). These additional rescue and enzymatic activity assays provided direct functional evidence that Aβ42‐induced impairment of Me3 catalytic activity contributes to mitochondrial dysfunction. Furthermore, we performed conformational simulation using the HDOCK server [35] and screened the optimal binding model with the highest matching degree with our HDX‐MS and CL(cross‐link)‐MS data. The results showed hydrogen bond interaction between Me3 (at residue sites R341, I343, and G488) and Aβ42 (at residue sites His13 and Asn27), indicating that these residue sites were key ones for Me3–Aβ42 binding (Figure 5E).

The above HDX‐MS and CL‐MS analyses identified Me3 as a potential Aβ42‐interacting protein. We next performed co‐immunoprecipitation (CoIP) assays to validate the Aβ42–Me3 interaction and assess its selectivity. Anti‐Aβ42 immunoprecipitation pulled down Me3, whereas IgG did not, supporting an interaction between Aβ42 and Me3 (Figure 5F). To further assess the selectivity of this interaction, we examined whether other malic enzyme isoforms or Aβ isoforms exhibited similar interactions. Anti‐Aβ42 immunoprecipitation did not pull down detectable Me1 or Me2, although both proteins were present in the input samples (Figure S10A). Moreover, when exogenous Aβ40 or Aβ42 peptides were added, Me3 was preferentially enriched in the Aβ42 group compared with the Aβ40 and IgG groups, whereas input Me3 levels were comparable (Figure S10B). Together, these results suggested that Aβ42 preferentially interacted with Me3 under the tested conditions, compared with Aβ40 and other malic enzyme isoforms.

Having biochemically supported the Aβ42–Me3 interaction, we further performed immunofluorescence (IF) assays of HT22 cells overexpressing Me3 and APP (Figure 5G). In Me3‐overexpressing cells, Me3 aggregation was observed, suggesting that Me3 overexpression itself may promote its aggregation. Compared to cells with no overexpression of APP, APP‐overexpressing cells exhibited marked aggregation and colocalization of Aβ42 and Me3, whereas no aggregation of Aβ42 or Me3 was observed in the APP‐overexpressing cells further treated with β‐Sheet Breaker Peptide iAβ5 (iAβ5) with high binding affinity towards Aβ42. This suggested that increased Aβ levels in mitochondria induced Me3 aggregation, thereby causing mitochondrial damage. After treatment with iAβ5, the expression levels of Park2 (mitophagy‐related protein) and the LC3‐II/LC3‐I ratio appeared lower than those in APP‐overexpressing cells without iAβ5 treatment, suggesting a potential trend toward the restoration of mitochondrial function (Figure S11).

### 2.6 Knockdown of Me3 Mitigates Mitochondrial Dysfunction and Mitophagy Impairment

**FIGURE 6:**
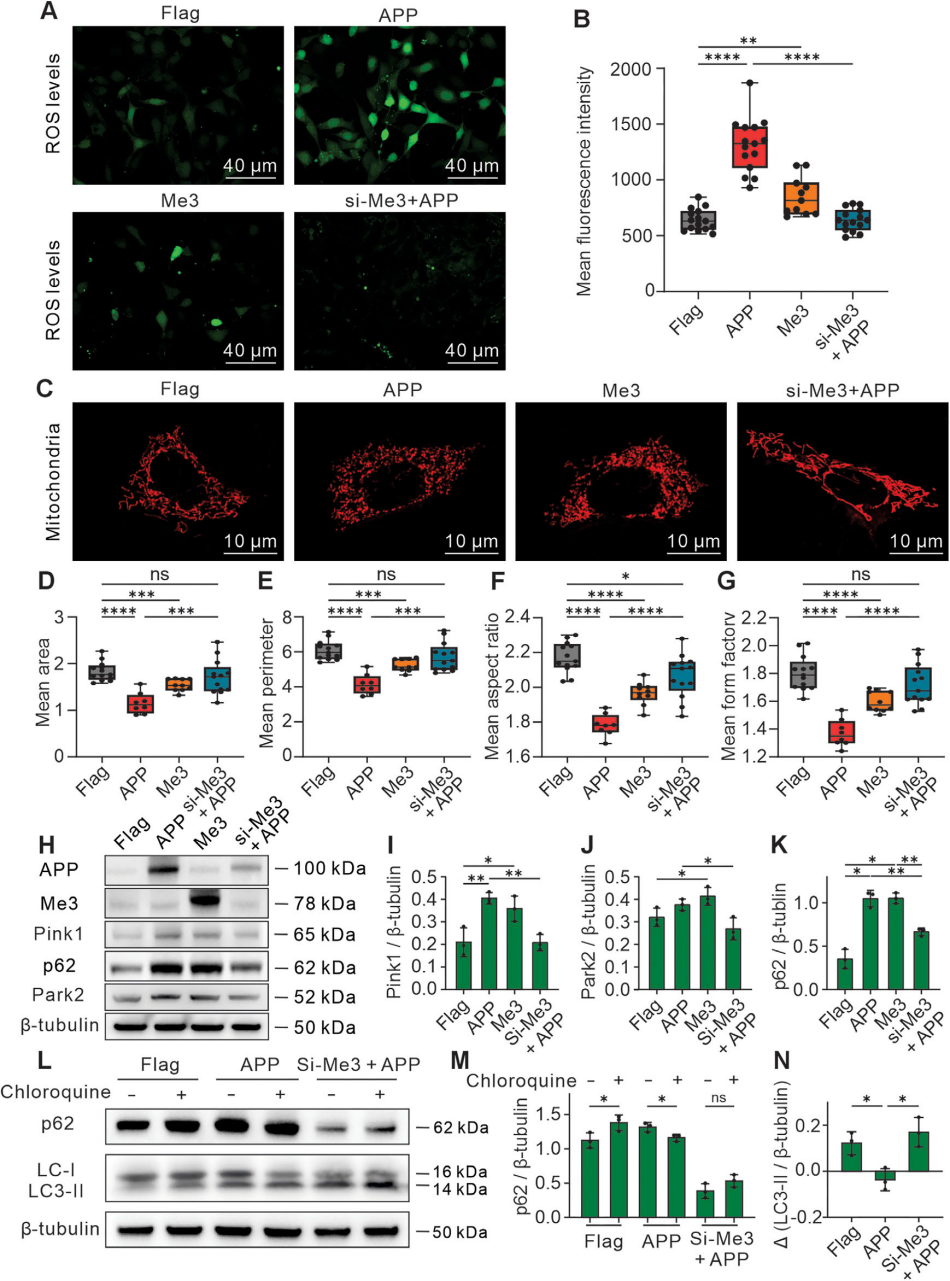
Me3 knockdown alleviates Aβ‐induced mitochondrial dysfunction, ROS accumulation, and mitophagy blockade. (A) DCFH‐DA staining fluorescence images of ROS level in HT22 cells transfected with four various plasmids. APP‐transfected cells (APP) exhibited significantly higher ROS levels than cells transfected with Flag control (Flag), whereas siRNA‐mediated Me3 knockdown (si‐Me3+APP) restored ROS levels. (B) Mean ROS fluorescence intensity across groups. (C) Mitochondrial fluorescence images in cells transfected with various plasmids. Me3 knockdown partially rescued mitochondrial fragmentation. (D‐G) Mitochondrial morphology parameters in different groups: mean mitochondrial area (D), perimeter (E), aspect ratio (F), and form factor (G) (*n* = 3, biological replicates). APP overexpression reduced mitochondrial size and elongation, consistent with mitochondrial fragmentation, whereas Me3 knockdown significantly reversed these defects. (H–K) WB analysis of mitophagy‐related proteins Pink1 (I), p62 (J), and Park2 (K). APP overexpression increased p62 accumulation, indicating impaired autophagic flux, while Me3 knockdown ameliorated these changes. (L–N) WB analysis of cells treated with chloroquine (CQ) to assess flux by measuring p62 (M) and LC3‐II (N) accumulation. All quantitative data were presented as mean ± SEM. Statistical significance was determined by Mann–Whitney test. ns, not significant; *p < 0.05; **p < 0.01; ***p < 0.001; ****p < 0.0001.

To elucidate the role of Me3 in AD pathogenesis, endogenous Me3 was knockdown in HT22 cells by specific small interfering RNA (si‐Me3) (Figure S12). Mitochondria are the primary intracellular source of ROS, and in turn, ROS dynamically affects mitochondrial function and mitophagy. In this study, we determined ROS level in HT22 cells with various treatments including Flag, APP, Me3, and APP after si‐Me3 knockdown (Figure 6A,B). Compared to the Flag treatment group, APP‐overexpressing cells exhibited significantly increased ROS level, consistent with the disruption of the TCA cycle induced by the overexpression of APP. In Me3‐overexpressing cells, there was a mild elevation in ROS level, indicating that Me3 aggregation impaired its function and further disrupted the TCA cycle. Notably, when APP was overexpressed in Me3‐knockdown cells, ROS level was significantly lower than that in cells with APP overexpression alone, indicating that Me3 knockdown mitigated Aβ‐induced TCA disruption.

Next, we evaluated mitochondrial morphology using MitoTracker staining and confocal imaging (Figure 6C). The morphological indicators for evaluating mitochondrial fragmentation, including area, perimeter, form factor, and aspect ratio, were calculated using the Mitochondria Analyzer Plugin in Fiji (ImageJ) software. The lower the indicator values, the more severe the mitochondrial fragmentation. APP‐overexpressing group exhibited the most severe mitochondrial fragmentation, followed by the Me3‐overexpressing group, which was more severe than the Flag group, but no significant differences in mitochondrial fragmentation degree were observed between the Me3‐knockdown group and Flag group (Figure 6C–G). These results further confirmed that Me3 aggregation induced mitochondrial impairment, and that Aβ‐Me3 co‐aggregation exerted more severe effects on mitochondrial impairment. Notably, even when APP was overexpressed in cells, Me3 knockdown maintained mitochondrial integrity well, suggesting that Me3 was crucial for repairing mitochondrial damage induced by Aβ.

Given the increased ROS level and fragmented mitochondrial morphology in APP‐overexpressing cells, mitophagy‐related protein expression levels were determined through WB analysis to evaluate impaired mitochondrial clearance. The WB results revealed that the levels of Pink1, Park2, and p62 were markedly upregulated in both APP‐ and Me3‐overexpressing cells, suggesting activation of the Pink1/Park2 mitophagy pathway accompanied by defective mitophagy degradation (Figure 6H–K). Meanwhile, those mitophagy‐related protein levels were significantly lower in the Me3‐knockdown group than in the APP‐ and Me3‐ overexpressing groups, demonstrating that Me3 was crucial for Aβ‐induced mitophagy impairment. Furthermore, cells were treated with chloroquine (CQ) to assess autophagic flux by inhibiting lysosomal degradation. CQ treatment increased p62 accumulation in control and APP‐overexpressing cells, whereas p62 levels were markedly lower in the Me3‐knockdown APP group (Figure 6L,M). In addition, the CQ‐induced change in LC3‐II level was reduced in APP‐overexpressing cells but restored with Me3 knockdown, indicating that APP‐induced impairment of mitophagic flux was largely dependent on Aβ‐Me3 co‐aggregation (Figure 6L,N). Collectively, we confirmed that free Aβs had a limited impact on mitochondrial homeostasis, whereas their co‐aggregation with Me3 initiated and enhanced mitochondrial dysfunction.

### 2.7 Knockdown of Me3 Mitigates Mitophagy Impairment in APP/PS1 Mice

**FIGURE 7:**
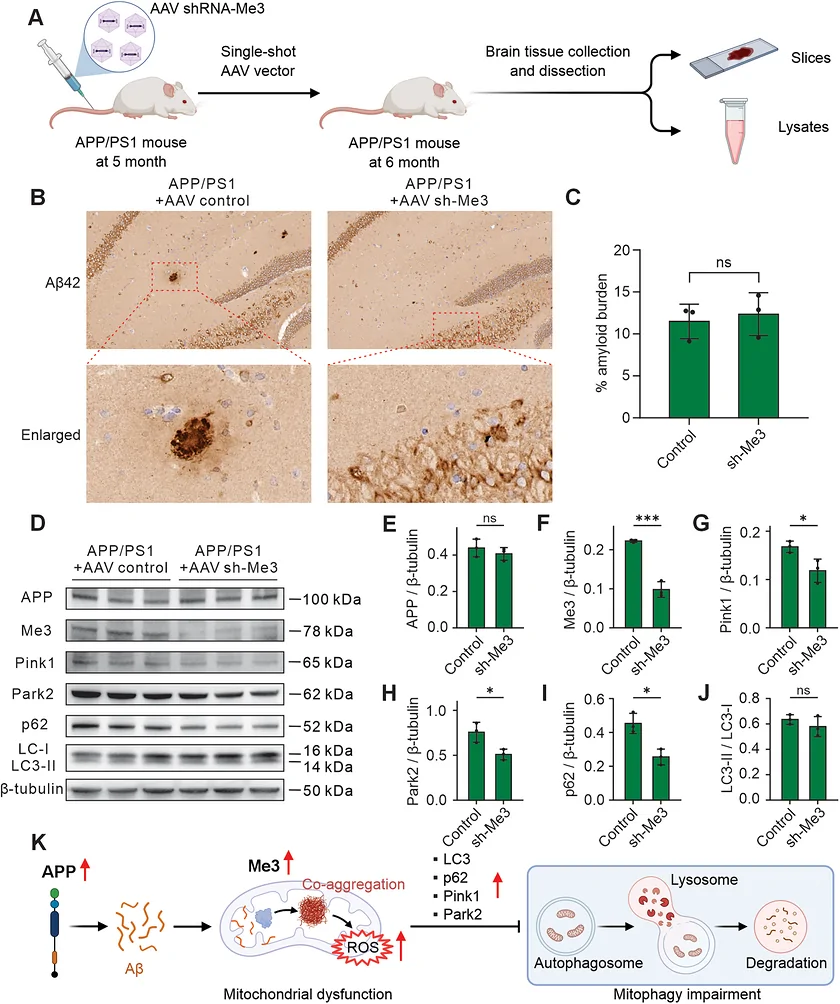
Me3 knockdown reduced the accumulation of mitophagy‐associated proteins in APP/PS1 mice. (A) Experimental design for adeno‐associated viruses (AAV)‐mediated Me3 knockdown in 5‐month‐old APP/PS1 mice. Brain tissues were collected one month after AAV injection for histological and biochemical analyses (created with BioRender.com). (B) Representative Aβ42 immunohistochemistry images of brain sections from APP/PS1 mice injected with AAV control or AAV‐shMe3. (C) Quantification of amyloid burden showed no significant difference (*p* = 0.400) between the control and shMe3 groups. (D) WB analysis of APP, Me3, Pink1, Park2, p62, LC3‐I, LC3‐II, and β‐tubulin in hippocampus. (E–J) Quantification of APP, Me3, Pink1, Park2, p62, and LC3‐II/LC3‐I levels. (K) Proposed model linking Me3 to Aβ‐associated mitochondrial dysfunction and mitophagy impairment (created with BioRender.com). All quantitative data were presented as mean ± SEM of three biological replicates. Statistical significance was determined by Mann–Whitney test. ns, not significant; *p < 0.05; ***p < 0.001.

To validate the pathological relevance of Aβ‐Me3 co‐aggregation in vivo, we knocked down Me3 in APP/PS1 mice using an AAV‐mediated shRNA intervention strategy. Intravenous injections of AAV‐sh‐Me3 or control virus were administered to 5‐month‐old APP/PS1 mice, and brain tissues were harvested at 6 months of age for pathological and protein expression analysis (Figure 7A). Immunohistochemical analysis with an Aβ42 antibody showed that the morphology and distribution of Aβ‐positive amyloid plaques in brain tissue from the sh‐Me3 group did not differ significantly from those in the control group. While the amyloid burden showed no significant difference (*p* = 0.4) between the two groups, a few larger plaques were observed in the control group, suggesting that Aβ aggregation was reduced (Figure 7B,C). We next conducted WB analysis to validate the role of Me3 in Aβ‐induced impairment of mitophagy. The WB results showed no significant difference in APP protein expression between the two groups, whereas Me3 protein expression was significantly reduced in the sh‐Me3 group, indicating effective viral intervention (Figure 7D–F). Notably, compared with the control group, the sh‐Me3 group showed significantly decreased expression of the mitophagy‐related proteins Pink1, Park2, and p62, while the LC3‐II/LC3‐I ratio did not differ significantly (Figure 7D,G–J). These results suggested that Me3 knockdown alleviated impairment of the Pink1/Park2‐mediated mitophagy, consistent with the above cell‐based results. Collectively, this work confirmed that Me3 enhanced the Aβ‐induced mitochondrial dysfunction in the brains of mice at the early stage of AD. A summary of the key results of this study was shown in the schematic in Figure 7K.

## 3 Discussion

AD pathogenesis is complex, and Aβ aggregation and mitochondrial dysfunction are two typical pathological features of AD in its early stages [16]. However, the molecular mediators linking these two events have yet to be fully elucidated. In this study, we identified Me3 as a potential mediator bridging Aβ42 accumulation with mitochondrial metabolic disturbance and mitophagy impairment during early AD pathology. Our proteomic and ultrastructural analyses indicated that mitochondrial dysfunction emerges early in APP/PS1 mice, which overexpress APP. Mitochondria‐related pathways, including oxidative phosphorylation, pyruvate metabolism, and autophagy, were prominently altered, accompanied by mitochondrial swelling and cristae disruption (Figures 1 and 2). These findings supported the concept that mitochondrial impairment is not merely a downstream consequence of neurodegeneration but actively participates in early AD progression. Consistently, APP overexpression disrupted mitochondrial energy metabolism, as evidenced by reduced levels of glycolytic intermediates and ATP, along with accumulation of glutamine and succinate (Figure 3). This metabolic profile suggested impaired glycolytic flux into the TCA cycle and succinate‐associated mitochondrial stress, both of which may fuel ROS generation and mitochondrial dysfunction.

Among TCA cycle‐related proteins, Me3 was consistently upregulated in early AD groups. As a mitochondrial NADP^+^‐dependent malic enzyme, Me3 contributes to pyruvate metabolism and redox homeostasis. Its upregulation might therefore represent an initial compensatory response to metabolic stress [25, 28]. However, in the context of Aβ42 accumulation driven by APP overexpression, our data suggested that this response becomes maladaptive. Multiple complementary approaches supported a preferential interaction between Aβ42 and Me3, as evidenced by colocalization analysis, HDX‐MS, CL‐MS, molecular docking, and co‐immunoprecipitation (Figure 5). This interaction likely promoted the formation of Me3‐containing co‐aggregates within mitochondria and interfered with Me3 function. Mechanistically, Aβ42–Me3 co‐aggregation was associated with increased oxidative stress, mitochondrial fragmentation, and impaired mitophagy. Me3 knockdown alleviated these mitochondrial abnormalities in APP‐overexpressing cells and reduced mitophagy‐related markers in APP/PS1 mice, positioning Me3 downstream of Aβ production and primarily linked to mitochondrial damage and defective quality control (Figures 6 and 7).

Nonetheless, the therapeutic implications of targeting Me3 warrant caution. Complete suppression of Me3 could impair mitochondrial metabolism, weaken antioxidant capacity, or disrupt adaptive responses to metabolic stress [9, 27, 29]. A more selective and potentially safer approach may be to disrupt the pathological Me3–Aβ42 interaction rather than broadly suppress Me3 expression or enzymatic activity. The Aβ42‐binding regions identified by HDX‐MS and CL‐MS offer a structural basis for developing molecules that selectively disrupt the Me3–Aβ42 interface while preserving normal Me3 function. Moreover, the β‐sheet breaker peptide iAβ5 reduced Aβ42–Me3 co‐aggregation and partially improved mitochondrial phenotypes, supporting the concept that limiting pathological co‐aggregation is preferable to global Me3 inhibition.

Several limitations should be acknowledged. This study focused primarily on early AD‐like pathology in APP/PS1 mice, a stage at which mitochondrial stress responses and compensatory protein upregulation are still detectable. By contrast, mitochondrial biogenesis and mitochondria‐related proteins are often broadly downregulated in symptomatic AD patients, likely reflecting a later stage of mitochondrial failure [36]. Whether Me3 upregulation and Me3–Aβ42 co‐aggregation persist across different disease stages, particularly in clinically manifest AD, remains to be determined. Additionally, the APP/PS1 model mainly recapitulates Aβ‐driven pathology and does not fully replicate the complexity of sporadic AD. Whether selective disruption of the Me3–Aβ42 interaction can improve neuronal function and cognition requires further validation. Future studies employing additional AD models, human‐derived systems, clinical samples spanning different disease stages, and long‐term functional assessments will be essential to firmly establish the pathological and therapeutic relevance of the Me3–Aβ42 network.

In conclusion, our study identified Me3 as a molecular link between Aβ42 accumulation, mitochondrial dysfunction, and impaired mitophagy in early AD models. Rather than advocating indiscriminate Me3 inhibition, these findings highlight the pathological Me3–Aβ42 interaction as a more precise therapeutic target. Selectively disrupting Me3–Aβ42 co‐aggregation while preserving the physiological metabolic functions of Me3 may offer a more balanced strategy to mitigate mitochondrial dysfunction in early AD.

## 4 Materials and Methods

### 4.1 Collection of Mouse Brain Tissue Samples and Protein Extraction

Three‐month‐old (approximately 12 weeks old) and 6‐month‐old (approximately 24 weeks old) APP/PS1 mice (male), as well as C57BL/six mice (male) with four biological replicates per group, were purchased from Huachuang Sino PharmaTech Co. Ltd. (Taizhou, China). Cortical and hippocampal tissues of mice were collected with reference to the Allen Reference Atlas. Mouse brain tissue homogenates were prepared as follows. Briefly, approximately 20 mg of tissue was added with zirconia beads (from Gene Company, China) at a ratio of 1:1 (volume) and 200 µL of radio immunoprecipitation assay (RIPA) lysis buffer containing 1 mM phenylmethanesulfonyl fluoride (PMSF) (Beyotime Biotechnology, China). The mixture was homogenized in a tissue cell disruptor (Bullet Blender Storm Pro; Gene Company, China) with the speed set as level 8 for 3 min. After homogenization, the supernatant was collected, and the precipitate was added with an additional 200 µL of fresh RIPA lysis buffer and homogenized once again. The supernatants collected from two times of homogenization were mixed. The mixed supernatant was centrifuged at 5000 × *g* and 4°C for 15 min. The resulting supernatant was collected, and its protein concentrations were measured using the bicinchoninic acid (BCA) assay. The supernatant was stored at −80°C until subsequent use.

### 4.2 Protein Digestion and Peptide Purification

Protein digestion was conducted following the FASP protocol. Specifically, 100 µg of extracted protein was diluted in 200 µL phosphate‐buffered saline (PBS), transferred to 1.5 mL ultrafiltration units (3 kDa, Millipore) (Merck, America), centrifuged at 12,000 × *g* and 4°C for 15 min, and added with 200 µL of 8 M urea containing 10 mM dithiothreitol (DTT) and 50 mM ammonium bicarbonate (ABC) (Sigma, America). The mixture was centrifuged at 12,000 × *g* and 20°C for 15 min, and these urea‐addition and centrifugation steps were repeated twice. Subsequently, the sample was treated with 200 µL of 8 M urea (10 mM DTT, 50 mM ABC), incubated at 37°C for 2.5 h, and supplemented with 10 µL of 1 M iodoacetamide (IAA; Sigma, America), followed by a 30‐min alkylation reaction in the dark. After alkylation, the sample was centrifuged at 12,000 × *g* and 20°C for 15 min, treated with 200 µL of 50 mM ABC, and centrifuged again (12,000 × *g*, 15 min, 20°C) to remove high‐concentration urea, with the urea removal step repeated twice. The sample was collected, treated with 100 µL of 50 mM ABC and 5 µL trypsin (0.4 µg/µL; Promega, America), and incubated at 37°C overnight. Afterwards, digested peptides were collected by centrifugation (12,000 ×* g* and 4°C for 15 min). To maximize peptide recovery, centrifugation (12,000 × *g*, 15 min, 4°C) was repeated twice, with 100 µL of ultrapure water added to the ultrafiltration unit twice, and all peptide filtrates were pooled. The obtained peptide solution was acidified until pH = 3 with 10% trifluoroacetic acid (TFA; Sigma, America). Subsequently, the peptide solution was desalted using C18 solid‐phase extraction (SPE) columns (Waters, America). Specifically, the C18 columns were first activated with methanol, then equilibrated with ultrapure water containing 0.1% TFA. Then, the acidified peptide solution was loaded onto the equilibrated columns, passing through the column until complete filtration. The columns carrying peptides were washed with 0.1% TFA to remove unbound impurities, and finally peptides were eluted from the columns with 50% acetonitrile (ACN; Merck, America) containing 0.1% TFA and collected into new microcentrifuge tubes. The collected peptides were vacuum‐dried and stored at −20°C for subsequent analysis.

### 4.3 Data Acquisition With Mass Spectrometer

The lyophilized peptide powder was redissolved in 0.1% formic acid (FA; Thermo Scientific, America). To separate different types of peptides, 200 ng of the resulting peptide solution was injected to nanoElute liquid chromatography (LC) system (Bruker Daltonics, America) equipped with an Inopticks C18 column (250 mm × 75 µm). The chromatographic conditions were as follows: Mobile phase A was water containing 0.1% FA, mobile phase B was acetonitrile containing 0.1% FA, a flow rate was set as 300 nL/min, and the total analysis duration was 1 h. The gradient program of mobile phase B was as follows: 2%–22% phase B from 0 to 45 min, 22%–37% phase B from 45 to 50 min, 37%–80% phase B from 50 to 55 min, followed by 80% phase B maintenance for 5 min. All fractions were subsequently analyzed using a TIMS‐TOF Pro mass spectrometer (Bruker Daltonics, America). The mass spectrometry parameters were set as follows: scan range of 100–1700 *m/z*, mobility range of 0.7–1.3 V s/cm^2^, cycle time per acquisition of 1.16 s, a scan mode involving one full MS scan followed by 10 PASEF (parallel accumulation‐serial fragmentation) MS/MS scans, intensity threshold of 5000, accumulation and release time of 100 ms, ion source voltage of 1500 V, auxiliary gas flow rate of 3 L/min, and ion source temperature of 180°C.

### 4.4 Mass Spectrometry Data Processing and Analysis

All raw mass spectrometry data were imported into FragPipe software for data retrieval and label‐free quantification (LFQ) analysis, and the mouse (*Mus musculus*) proteome reference database from the UniProt Swiss‐Prot database was used. Key retrieval parameters were set as follows: Variable modifications included oxidation (M) and acetylation (Protein N‐terminus); the fixed modification was carbamidomethylation (C); and Trypsin/P was selected as the digestion enzyme. The retrieval results with a false discovery rate (FDR) < 1% were retained at both the protein and peptide levels, and the obtained clean data were used for subsequent analyses. The protein peak areas obtained from the retrieval were employed for statistical analysis. During data preprocessing, proteins with an overall missing value rate > 50% were excluded; the missing values of remaining proteins were imputed through a normal distribution (with a standard deviation shift of 1.8 and a standard deviation width of 0.25) based on the mass spectrometry detection limit of samples. A *t*‐test was performed using R packages, and a FC of ≥ 1.5 (upregulation) or ≤ 0.67 (downregulation) was set as the threshold for screening DEPs. GO and KEGG enrichment analyses were conducted via the Metascape and KOBAS web‐based tools, respectively, and the results were visualized using R packages.

### 4.5 Cell Culture and Transfection

The HT22 (Mouse hippocampal neurons) cells (Catalog: CL‐0697, RRID: CVCL_0321, STR verified) were purchased from Wuhan Procell Life Science & Technology Co. Ltd. (Wuhan, China). HT22 cells were determined to be free of mycoplasma contamination and were cultured in Dulbecco's modified Eagle medium (DMEM) containing 10% fetal bovine serum (FBS) (Gibco, America) in an incubator at 37°C with 5% CO_2_. The cell culture was trypsinized at room temperature for 1–2 min, and then 1/4 to 1/2 cells were subjected to continuous culture. Plasmids Flag (Catalog: P45080), APP (Catalog: P51694), Me3 (Catalog: P64080), and Me3‐R190A/N443A were supplied by Miaoling Biotechnology Co. Ltd. (Wuhan, China). When cell confluency reached approximately 70%, cell transfection was carried out following the manufacturer's instructions of Lipofectamine 3000 (Thermo Scientific, America) to overexpress target proteins. Specifically, 1 µg of plasmid, 2 µL of P3000 reagent, and 2 µL of Lipofectamine 3000 were added into serum‐reduced medium (Thermo Scientific, America), mixed, and incubated for 10 min. The incubated mixture was added dropwise to cell culture flasks/dishes containing freshly replaced DMEM and cultured for an additional 24 h for subsequent analyses. Si‐Me3 (small interfering RNA) was purchased from RiboBio Co. Ltd. (Catalog: siB1122593042‐1‐5, Guangzhou, China). After cell confluency reached approximately 30%, HT22 cells were transfected with Si‐Me3 for 48 h and further transfected with the APP plasmid.

### 4.6 Targeted Metabolomics Analysis

HT22 cells transfected with Flag and APP plasmids were collected and washed three times with PBS. Subsequently, the resulting cell precipitate was added with 100 µL of ultrapure water and resuspended thoroughly to obtain a 100 µL cell suspension. For metabolite extraction, a 50 µL aliquoted cell suspension was added with 200 µL of prechilled (−20°C) methanol, vortexed at 2500 rpm for 2 min, frozen rapidly in liquid nitrogen for 5 min, then transferred on ice, and thawed for 5 min, followed by vortexing for 2 min to mix thoroughly. This freeze‐thaw‐vortex cycle was repeated for three times. The mixture was centrifuged at 12,000 rpm and 4°C for 10 min, and 200 µL of the supernatant was aspirated into a new centrifuge tube. Afterwards, the supernatant was incubated at −20°C for 30 min and centrifuged again at 12,000 rpm and 4°C for 10 min, and then 180 µL of the resulting supernatant was stood in a protein precipitation plate and stored at −20°C for subsequent mass spectrometric analysis. The remaining 50 µL of the cell suspension was subjected to three repeated freeze‐thaw‐vortex cycles in liquid nitrogen and centrifugation at 12,000 rpm for 10 min. The supernatant was collected for protein concentration determination through the BCA assay.

Data acquisition was performed using an ultra‐high‐performance liquid chromatography‐tandem mass spectrometry (UPLC‐MS/MS) system, consisting of a Waters ACQUITY H‐Class UPLC and a QTRAP 6500+ mass spectrometer. Chromatographic separation was carried out using an ACQUITY UPLC BEH Amide column (1.7 µm, 100 mm × 2.1 mm i.d.). Mobile phase A was ultrapure water containing 10 mM ammonium acetate and 0.3% ammonia solution, and mobile phase B was 90% acetonitrile‐containing water. The UPLC‐MS/MS parameters were as follows: flow rate of 0.40 mL/min, column temperature of 40°C, and injection volume of 2 µL. The gradient program of mobile phase B was 95% B from 0.0 to 1.2 min, declining to 70% B from 1.2 min to 8.0 min, further reducing to 50% B from 8.0 to 9.0 min, holding at 50% B from 9 min to 11 min, and rising to 95% B from 11.1 to 15 min. The mass spectrometer was equipped with an electrospray ionization (ESI) source at an ion source operating temperature of 550°C, exhibiting a spray voltage of 5500 V in positive ion mode and −4500 V in negative ion mode. The curtain gas (CUR) pressure was set as 35 psi, and each ion pair was scanned using the QTRAP 6500+ mass spectrometer with optimized parameters of declustering potential (DP) and collision energy (CE).

### 4.7 Transmission Electron Microscopy Analysis

Tissue sections (1–2 mm^3^) were fixed in 2.5% glutaraldehyde (Solarbio, China) and rinsed for three times with PBS. Subsequently, the sections were fixed in 1% osmium tetroxide (dissolved in PBS; Merck, America) at room temperature for 15 min, with the second fixation step repeated for six times. The samples were then rinsed with PBS for three times, with each rinse lasting 15 min, and dehydrated with seven various concentrations of ethanol (30%, 50%, 70%, 80%, 85%, 90%, and 100%), with each dehydration step lasting 15 min and 100% ethanol dehydration repeated twice. The resulting samples were infiltrated sequentially using acetone solution at the acetone/epoxy resin ratio of 2:1, then at acetone/epoxy resin ratio of 1:1, and pure epoxy resin (Merck, America) as infiltrants at 37°C incubator, with each infiltration step lasting for 8–12 h. After infiltration, the samples were transferred to molds, embedded in epoxy resin medium, and polymerized in a 60°C incubator for 48 h. The samples were cut into ultrathin sections (80–100 nm thick) with an ultramicrotome, followed by uranyl‐lead double staining including 15‐min staining with 2% uranyl acetate saturated aqueous solution at room temperature and 15‐minute staining with 40 mM lead citrate at room temperature. After double staining, the ultrathin sections were air‐dried overnight at room temperature and examined using a transmission electron microscope HT7800 (Hitachi).

### 4.8 Multiplex Immunohistochemical Analysis

Multiparametric IF staining was performed with a four‐color multiplex IF immunohistochemistry (mIHC) staining kit (Catalog: RS0035, ImmunoWay Biotechnology) using the tyramide signal amplification (TSA) technology, strictly following the manufacturer's instructions. Primary antibodies were diluted with immunol staining primary antibody dilution buffer (Beyotime Biotechnology, China), including Me3 (1:200, Abclonal, Catalog: A15787), β‐Amyloid(1‐42) (1:200, Abclonal, Catalog: A24422), LC3 (1:200, Proteintech, Catalog: 14600‐1‐AP), TOM20 (1:200, Proteintech, Catalog: 11802‐1‐AP), and LAMP1 (1:200, Proteintech, Catalog: 21997‐1‐AP). After staining, the sections were mounted with anti‐fade mounting medium, and the fluorescence images were acquired using a confocal laser scanning microscope. The acquired images were processed and analyzed using ImageJ software.

### 4.9 Immunofluorescence Analysis

HT22 cells were seeded into confocal dishes, cultured until confluency reached 70%, and then transfected with Me3 and APP plasmids in DMEM. After 24‐h transfection, the medium was discarded. The cells were washed twice with PBS, fixed in 500 µL of 4% paraformaldehyde (Beyotime Biotechnology, China) at room temperature for 15 min, rewashed with PBS for three times, and blocked with 500 µL of blocking buffer containing 0.5% bovine serum albumin (BSA) and 0.3% Triton X‐100 (Sigma, America) at room temperature for 1 h. After discarding the blocking buffer, primary antibodies (including Me3 [1:200, Abclonal, Catalog: A15787] and beta‐Amyloid(1‐42) [1:200, Bioss, bsm‐4713M]) were diluted with phosphate buffered saline with Tween 20 (PBST) containing 0.2% BSA and 0.3% Triton X‐100 at the dilution ratio of 1:200. The cells were added with 200 µL of diluted primary antibody, incubated at room temperature for 1 h, washed three times with PBS to remove the primary antibody solution. Next, the resulting cells were added with 200 µL of diluted fluorophore‐conjugated secondary antibody (with a dilution ratio of 1:200, Beyotime Biotechnology), incubated at room temperature for 1 h, and rewashed with PBS three times to remove the secondary antibody. Finally, the obtained cells were added with 1 mL of PBS to maintain their hydration and transferred to a confocal laser scanning microscope for observation and image acquisition.

### 4.10 Western Blot Analysis

To examine protein expression, WB analysis was performed. Briefly, 30 µg of total protein solution was diluted to 20 µL with PBS and treated with 5 µL of sodium dodecyl sulfate (SDS) loading buffer. The 25 µL mixture solution was boiled at 95°C for 10 min and centrifuged at 12,000 rpm for 5 min, and then the supernatant was collected and loaded onto the 4%–20% gradient gel for SDS‐polyacrylamide gel electrophoresis (SDS‐PAGE). After electrophoresis, proteins were transferred onto a polyvinylidene fluoride (PVDF) membrane via wet transfer. The PVDF membrane was washed with Tris‐buffered saline containing 0.1% Tween‐20 (TBST) for three times (5 min per time), blocked with a rapid blocking solution at room temperature for 20 min, and rewashed with TBST to remove the blocking solution. The rewashed PVDF membrane was incubated at 4°C overnight respectively with primary antibodies diluted with PBS (containing 0.2% BSA and 0.3% Triton X‐100), including Me3 (1:1000, Abclonal, Catalog: A15787), SQSTM1/p62 (1:3000, Abclonal, Catalog: A7758), APP/Beta Amyloid (1:1000, Proteintech, Catalog: 25524‐1‐AP), LC3 (1:4000, Proteintech, Catalog: 14600‐1‐AP), Me1 (1:2000, Proteintech, Catalog: 16619‐1‐AP), Me2 (1:1000, abinScience, Catalog: HB858014), Beta Tubulin (1:6000, Proteintech, Catalog: 10094‐1‐AP), and GAPDH (1:20,000, Proteintech, Catalog: 10494‐1‐AP). The primary antibody‐incubated membrane was washed three times with TBST (5 min per time), incubated with diluted horseradish peroxidase (HRP)‐conjugated secondary antibody solution (1:5000, Proteintech, Catalog: SA00001‐2) at room temperature for 1 h, and rewashed with TBST for three times (5 min per time) to discard the secondary antibody solution. Equal volumes of enhanced chemiluminescence (ECL, Proteintech, Catalog: PK10003) reagent solution A and solution B were mixed, and 2 mL of this mixture solution was added dropwise to the membrane. Protein bands were detected using a chemiluminescence imaging system.

### 4.11 Cycloheximide Chase Assay

HT22 and APP‐overexpressing cells were treated with CHX (50 µg/mL) to inhibit new protein synthesis. Cells were harvested at 0, 12, 16, and 20 h after CHX treatment. Total proteins were extracted using RIPA lysis buffer containing PMSF protease inhibitors, and protein concentrations were determined using a BCA assay. Equal amounts of protein were subjected to WB analysis using antibodies against Me3 and β‐tubulin.

### 4.12 Co‐Immunoprecipitation Assay

CoIP was performed to validate the interaction between Aβ and Me proteins. Cells were lysed in ice‐cold IP lysis buffer supplemented with a protease inhibitor cocktail (Thermo Scientific), and the lysates were clarified by centrifugation at 4°C. A portion of each lysate was retained as input. Equal amounts of protein were incubated with anti‐Aβ42 antibody or IgG overnight at 4°C. The immune complexes were then captured using Protein A/G magnetic beads (Thermo Scientific) according to the manufacturer's instructions. After incubation, the beads were collected using a magnetic stand and washed several times with cold lysis buffer. The bound proteins were boiled in SDS loading buffer and subjected to WB analysis.

### 4.13 Hydrogen Deuterium Exchange Mass Spectrometry (HDX‐MS) Analysis

The 100 µM Me3 samples (Cusabio, Catalog: CSB‐EP613700HU, China) and Me3 (100 µM)‐Aβ42 (Beyotime Biotechnology, Catalog: P9001, China) solution samples at molar ratios of 1:2 were prepared and equilibrated for 1 h. HDX‐MS was performed to examine the peptide level in both samples. Both samples were diluted with equilibrium buffer containing 100 mM sodium phosphate (pH 7.0) to a final concentration of 20 µM Me3. Briefly, at the start of an HDX reaction (time zero, *t* = 0), 20 µM protein solution was added into labeling buffer (containing 100 mM sodium phosphate dissolved in D_2_O, pH 6.6) at a ratio of approximately 1:20 (volume) and incubated at 20°C. The hydrogen‐deuterium exchange was quenched by adding chilled quenching buffer (100 mM sodium phosphate, pH 2.0) at the ratio of 1:1 at 10 s, 1 min, 10 min, 30 min, and 60 min post reaction. Quenched samples were digested, desalted, and separated using an Acquity UPLC M‐Class system with HDX automation coupled with Synapt XS HDMS. The protein was digested using an immobilized pepsin column (2.1 mm × 30 mm, Enzymate Pepsin Column; Waters, America) of the HDX manager at 20°C for 5 min in 0.1% formic acid, with a flow rate of 40 µL/min of column compartment. The obtained peptides were collected and desalted by a trap column (ACQUITY UPLC BEH C18 VanGuard pre‐column, 130 Å, 1.7 µm, 2.1 mm × 5 mm; Waters, America), and subsequently separated with an Acquity UPLC BEH C18, 130 Å, 1.7 µm, 1 mm × 100 mm column (Waters, America) with column temperature held at 0°C. After 7‐min gradient elution, the resulting fraction was imported into a Synapt XS HDMS. Mass spectra of peptides were acquired in the elevated mass spectrometry (MS^E^) mode within a mass range of 100–2000. Then, peptide sequences were identified using ProteinLynx Global Server (Waters), and the deuterium uptake number at different time points was calculated using DynamX 3.0 (Waters).

### 4.14 Chemical Cross‐Linking

Me3 (15 mM) and Aβ42 (30 mM) were co‐incubated at room temperature for 60 min before initiating the cross‐linking reactions. The incubated protein mixture was treated with 1.5 mM bis (sulfosuccinimidyl) suberate (BS3) solution (Thermo Scientific, America) as a cross‐linker and incubated at 25°C with spinning at 500 rpm for 45 min. After a 15‐minute incubation, Tris‐HCl (1 µL of 1 M, pH 7.4) was added to quench the cross‐linking reaction.

### 4.15 Me3 Enzymatic Activity Assay

Me3 enzymatic activity was determined using an NADP‐malic enzyme activity assay kit (Solarbio, China) according to the manufacturer's instructions. HT22 cells were collected after the indicated transfections and washed with cold PBS. Cell pellets were lysed in extraction buffer and sonicated on ice. The lysates were centrifuged at 8000 *g* for 10 min at 4°C, and the supernatants were collected for enzyme activity measurement. Protein concentrations were determined using a BCA assay. Enzyme activity was calculated from the change in absorbance and normalized to protein concentration. Results were expressed as U/mg protein.

### 4.16 Determination of ROS and Mitochondria

HT22 cells were cultured in confocal dishes and transfected for 24 h for ROS detection and mitochondrial imaging analysis. Intracellular ROS level was quantified using 2',7'‐dichlorodihydrofluorescein diacetate (DCFH‐DA; Yeasen Biotechnology, China). Mitochondria were stained with Mito‐Tracker Red CMXRos (Beyotime Biotechnology, China), strictly following the manufacturer's instructions. After staining, the cells were transferred to a confocal laser scanning microscope for image acquisition. All acquired images were analyzed using ImageJ software.

### 4.17 AAV‐Mediated Me3 Knockdown in APP/PS1 Mice

Five‐month‐old (approximately 20 weeks old) APP/PS1 mice (male) were obtained from Huachuang Sino PharmaTech Co., Ltd. (Taizhou, China) and housed under standard laboratory conditions with controlled temperature and humidity, a 12‐h light/dark cycle, and free access to food and water. Mice were randomly assigned to the AAV‐control group or AAV‐shMe3 group, with *n* = 3 mice per group. Mice received a single tail vein injection of AAV‐control or AAV‐shMe3 (3.3 × 10^11^ vg per mouse). After viral administration, mice were maintained for 1 month to allow sufficient viral expression and Me3 knockdown. At 6 months of age (approximately 24 weeks old), mice were deeply anesthetized, and brain tissues were collected for histological and biochemical analyses. All animal experiments were reviewed and approved by the Animal Research Management Committee of Huazhong University of Science and Technology (IACUC No. 4151). The study was conducted in accordance with the 3Rs principles (Replacement, Reduction, and Refinement), and the animal experiments were reported in compliance with the ARRIVE guidelines.

## Author Contributions

W.Z. and Y.Z. contributed equally to this work. Conceptualization: X.L. and Y.L. Methodology: X.L., Y.L., and W.Z. Visualization: W.Z., Y.Z., and P.L. Validation: P.L. and Y.Z. Software: W.Z. and Y.Z. Funding acquisition and project administration: X.L. Supervision: Y.L. Writing – original draft: W.Z. and Y.Z. Writing – review and editing: X.L., Y.L., and W.Z. All authors have read and approved the final manuscript.

## Funding

This work was supported by the National Natural Science Foundation of China (Grant Nos. 81827901 and U25A20562).

## Ethics Statement

All animal experiments were reviewed and approved by the Animal Research Management Committee of Huazhong University of Science and Technology (IACUC No. 4151). All animal procedures were conducted in accordance with the 3Rs principles (Replacement, Reduction, and Refinement) and reported in compliance with the ARRIVE guidelines for animal research.

## Conflicts of Interest

The authors declare no conflicts of interest.

## Supporting information




**Supporting file 1**: mco270984‐sup‐0001‐SuppMat.docx

**Supporting file 2**: mco270984‐sup‐0002‐Table.xlsx

## Data Availability

The proteomics mass spectrometry data generated in this study have been deposited in the iProX database under accession number IPX0013745001. Other data supporting the findings of this study are included in the article and Supporting Information or are available from the corresponding author upon reasonable request.
