# Supplementary material for: Multi‐Omics Analysis Reveals That Aβ42‐ Me3 Co‐Aggregation Induces Mitophagy Blockade in Early Stages of Alzheimer's Disease
Source: MedComm (2020). 2026 Sep 8;7(9):e70984. doi: 10.1002/mco2.70984 (PMC13551346; doi:10.1002/mco2.70984)
Supplement: Supplementary file 1 [file MCO2-7-e70984-s002.docx]

Supplementary Information

Multi-omics analysis reveals that Aβ42- Me3 co-aggregation induces mitophagy blockade in early stages of Alzheimer’s disease

Wenjie Zhu^1^, Yuanyuan Zhang^1^, Pengjie Li^1^, Yingying Zhong^1^, Yawei Lin^2,*^, Xin Liu^1,*^

^1^ The Key Laboratory for Biomedical Photonics of MOE at Wuhan National Laboratory for Optoelectronics-Hubei Bioinformatics and Molecular Imaging Key Laboratory, Systems Biology Theme, Department of Biomedical Engineering, College of Life Science and Technology, Huazhong University of Science and Technology, Wuhan 430074, China.

^2^ School of Chemistry, Chemical Engineering and Life Sciences, Wuhan University of Technology, Wuhan 430070, China.

* Corresponding author:

Xin Liu; E-mail: xliu@mail.hust.edu.cn

Yawei Lin; E-mail: linyawei2012@whut.edu.cn

Figure S1. (A) Subcellular localization of identified proteins. (B) Correlation analysis of 4 replicates in each group of mouse brain samples. Value is the correlation coefficient (R^2^).

Figure S2. Heatmap of 55 mitochondrial energy metabolites quantified by LC-MS/MS.

Figure S3. WB analysis of APP and Me3 in 3- and 6-month-old mouse hippocampus.

Figure S4. Proteomic quantification of Me proteins in AD mice and WB analysis of (A) Proteomic quantification of Me1, Me2, and Me3 protein abundance in AD-related and control samples, shown as log₂-transformed intensity. (B-D) Western blot validation showed that Me1 and Me2 protein levels were not significantly altered between NC and APP cells. (E) Me3 expression in HT22 cells under oxidative stress, endoplasmic reticulum stress, mitochondrial inhibition, and glycolytic inhibition conditions. (F) CHX chase analysis of Me3 protein stability in NC and APP cells.

Figure S5. WB analysis of mitophagy-related proteins in 6-month-old mouse hippocampus.

Figure S6. (A) mIHC fluorescence signals of LC3 (red), TOM20 (green), and Aβ (cyan). AD mice exhibited accumulation of LC3 and co-localization of LC3 and TOM20, consistent with mitophagosome formation. (B) mIHC fluorescence signals of lysosomal marker LAMP1 (red), TOM20 (green), and Aβ (cyan). Colocalization between LAMP1 and TOM20 indicated mitophagosome-lysosome fusion.

Figure S7. Relative deuterium uptake change by HDX-MS.

Figure S8. Crosslink peptide of Aβ42 and Me3 identified by CL-MS.

Figure S9. Validation of Me3 overexpression and mutant expression in HT22 cells.

Figure S10. Aβ selectively interacts with Me3 in HT22 cells. (A) Me1 and Me2 were not detectably enriched in anti-Aβ42 immunoprecipitates. (B) Me3 was detected in Aβ40- and Aβ42-immunoprecipitated complexes, with stronger enrichment in the Aβ42 group.

Figure S11. WB analysis of Park2 and LC3-I/II expression in HT22 cells transfected with Flag, APP, or APP co-treated with iAβ5.

(Note: Two independent samples of APP-overexpressing cells (APP-Group1 and APP-Group2) are included here to illustrate the consistency of the changes in Park2 and LC3-II. The GAPDH signal in APP-Group2 is relatively weaker than in the other lanes, likely due to a minor loading variation during sample preparation. This variation does not affect the relative migration or identity of the Park2 and LC3-II bands; the overall trend remains consistent between APP-Group1 and APP-Group2.This experiment was performed once and is intended as a preliminary observation of trend rather than a statistically validated conclusion.)

Figure S12. Western blot verification of Me3 knockdown by si-Me3 in HT22 cells.
